# RNA-seq based SNPs for mapping in *Brassica juncea* (AABB): synteny analysis between the two constituent genomes A (from *B. rapa*) and B (from *B. nigra*) shows highly divergent gene block arrangement and unique block fragmentation patterns

**DOI:** 10.1186/1471-2164-15-396

**Published:** 2014-05-23

**Authors:** Kumar Paritosh, Vibha Gupta, Satish K Yadava, Priyansha Singh, Akshay K Pradhan, Deepak Pental

**Affiliations:** Centre for Genetic Manipulation of Crop Plants, University of Delhi South Campus, Benito Juarez Road, New Delhi, 110021 India; Department of Genetics, University of Delhi South Campus, Benito Juarez Road, New Delhi, 110021 India

**Keywords:** *Brassica* species, RNA-seq, SNP, Linkage map, Comparative genomics, Evolution

## Abstract

**Background:**

*Brassica juncea* (AABB) is an allotetraploid species containing genomes of *B. rapa* (AA) and *B. nigra* (BB). It is a major oilseed crop in South Asia, and grown on approximately 6–7 million hectares of land in India during the winter season under dryland conditions. *B. juncea* has two well defined gene pools – Indian and east European. Hybrids between the two gene pools are heterotic for yield. A large number of qualitative and quantitative traits need to be introgressed from one gene pool into the other. This study explores the availability of SNPs in RNA-seq generated contigs, and their use for general mapping, fine mapping of selected regions, and comparative arrangement of gene blocks on *B. juncea* A and B genomes.

**Results:**

RNA isolated from two lines of *B. juncea* – Varuna (Indian type) and Heera (east European type) – was sequenced using Illumina paired end sequencing technology, and assembled using the Velvet *de novo* programme. A and B genome specific contigs were identified in two steps. First, by aligning contigs against the *B. rapa* protein database (available at BRAD), and second by comparing percentage identity at the nucleotide level with *B. rapa* CDS and *B. nigra* transcriptome. 135,693 SNPs were recorded in the assembled partial gene models of Varuna and Heera, 85,473 in the A genome and 50,236 in the B. Using KASpar technology, 999 markers were added to an earlier intron polymorphism marker based map of a *B. juncea* Varuna x Heera DH population. Many new gene blocks were identified in the B genome. A number of SNP markers covered single copy homoeologues of the A and B genomes, and these were used to identify homoeologous blocks between the two genomes. Comparison of the block architecture of A and B genomes revealed extensive differences in gene block associations and block fragmentation patterns.

**Conclusions:**

Sufficient SNP markers are available for general and specific -region fine mapping of crosses between lines of two diverse *B. juncea* gene pools. Comparative gene block arrangement and block fragmentation patterns between A and B genomes support the hypothesis that the two genomes evolved from independent hexaploidy events.

**Electronic supplementary material:**

The online version of this article (doi: 10.1186/1471-2164-15-396) contains supplementary material, which is available to authorized users.

## Background

Brassica species belonging to the U’s triangle [1] consist three diploids – *B. rapa* (AA), *B. nigra* (BB) and *B. oleracea* (CC), and their allopolyploids – *B. juncea* (AABB), *B. napus* (AACC) and *B. carinata* (BBCC). These are major oilseed and vegetable crops that are grown worldwide barring the tropics. This complex of crop species is of critical importance to global food and nutritional security, and extensive breeding programmes are in place to enhance their yield potential. Breeding research has received a major fillip from next-generation sequencing efforts, with genomic resources allowing for more involved molecular mapping and marker based introgressions [2–4]. These genomic resources allow a deeper understanding of the molecular mechanisms behind critical qualitative and quantitative traits.

*B. napus* is a major oilseed crop of Europe, Canada and China, and has received great attention [5]. The two constituent species of *B. napus*, namely *B. rapa* and *B. oleracea*, are important in their own right as they contain important vegetable crops [6, 7]. A representative type of *B. rapa*[8] and also of *B. oleracea*[9] has been sequenced. *B. juncea*, a major oilseed crop of the Indian sub-continent, has received much less attention [10]. Furthermore, little genetic or genomics work has been carried out on *B. carinata* and *B. nigra* as they are minor crops. However, they could be important sources of resistance to many biotic and abiotic stresses, and be of use for the improvement of more extensively grown Brassica species.

In India, *B. juncea* is grown on approximately 6–7 million hectares of land [11]. The crop is well adapted to cultivation in the dry land areas of north-western parts of India, and is grown during the winter season, thus taking advantage of residual soil moisture availability following the end of the rainy season (August or early September). Due to its high yield potential under low water availability, *B. juncea* is a potential crop for other regions of the world that have low moisture availability and mild winters.

*B. juncea* has two distinct gene pools: the Indian, and the east European gene pool [12]. The Indian gene pool has narrow genetic diversity [13, 14]. Despite this limitation, extensive efforts in pure line breeding have led to varieties with a yield potential of around 2.2 tons/hectare under protective irrigation. The east European gene pool shows more diversity at the molecular level [13]. Hybrids between Indian and east European types are heterotic for yield, with a potential of up to 2.6 tons/hectare. Numerous qualitative and quantitative traits have been mapped in a doubled haploid (DH) population developed from a cross between Varuna, a national check variety belonging to the Indian gene pool, and Heera, a Canola quality or ‘00’ line belonging to the east European gene pool [15–21]. The positive value traits in each of the two gene pools need to be fine mapped, and transferred to the pool with lower value. This will improve the two gene pools and facilitate the development of hybrids that outperform current hybrids in yield and quality.

We have carried out RNA-seq of Varuna and Heera lines and used the sequence data to develop SNP markers for genome wide and specific-region fine mapping, following methods described earlier for oleiferous lines of *B. rapa*[22]. It is well established that the three diploids of U’s triangle, *B. rapa*, *B. nigra*, and *B. oleracea* evolved through a genome triplication event, described as the ***b*** event [23]. However, extensive gene fractionation has led to some genes being present as a single copy, some as two paralogues, and some as three paralogues in the diploid genome species [8, 24, 25]. As an example, for *B. rapa* the BRAD database lists 17,562 single copy gene models, 13,506 two copy and 6,645 three copy paralogous gene models [26, 27]. There are adequate SNPs available in the single-copy gene models of *B. rapa*, even in closely related lines, for genome wide mapping [22]. However, the availability of SNPs needs to be assessed in the allotetraploid *B. juncea*. It is expected that most single-copy genes of *B. rapa* (A genome) will have a homoeologue(s) in the B genome. As a consequence, both HSVs (homoeologue specific variations) and allele specific SNPs are required to be identified for the development of co-dominant markers for each locus.

We report that there are enough SNPs available between Varuna and Heera to identify homoeologues between A and B genomes, and paralogues within A and B genomes for both genome wide and specific -region fine mapping. Contigs obtained from RNA-seq data of *B. juncea* have been separated into A and B genome specific gene models by comparison with the BRAD database for the A genome and to our unpublished work on the RNA-seq of *B. nigra* for the B genome. The transcriptome based contig assemblies have been used to identify SNPs, and an extensive molecular map has been developed using DH lines derived from a Heera and Varuna cross. This map has provided new insights into the arrangement of A to X blocks of *A. thaliana*[28] in the A and B genomes of *B. juncea*.

Comparative mapping between A and C genomes and the model crucifer *A. thaliana* was first performed in *B. napus* (AACC) [29]. Based on the gene collinearity between A and C genomes in relation to *A. thaliana,* it was proposed that the *A. thaliana* genome could be represented as A to X gene blocks [28]. Each gene block of *A. thaliana* was shown to be represented as three copies in both the A and C genomes of *B. napus*, with high levels of collinearity in gene arrangement in the blocks [30, 31]. We found similar block triplication in the B genome of *B. juncea* using intron-polymorphism (IP) markers when comparing block organization in the A and B genomes of *B. juncea*[32]. The addition of 999 SNP markers to the earlier map [32] has allowed identification of new blocks in the B genome in the present study. Comparative mapping between the A and B genome reveals that none of the linkage groups (LGs) of the two genomes are homoeologous along their entire length, and that there are variations in the fragmentation pattern of some of the blocks. We discuss the implications of the gene block arrangements and block fragmentation patterns discerned in this study to the evolutionary relationship of A and B genomes.

## Results

### Transcriptome assembly

RNA isolated from various tissues of the two *B. juncea* lines Heera and Varuna, was used for transcriptome sequencing. Libraries were sequenced using a Genome Analyzer IIx instrument (Illumina), with 134,372,556 raw sequence reads obtained for Heera and 172,720,474 for Varuna. These sequences were filtered for low-quality reads. Additionally, 31 bases with low quality values were trimmed from the 3′ end of each of the sequences, following parameters described earlier [22]. This resulted in 128,745,616 and 134,116,723 paired-end sequences of 70 bp length for Heera and Varuna, respectively (Additional file 1). Assembly of the cleaned sequences was carried out using the Velvet *de novo* assembly program at K-mer values ranging from 21 to 57, at an interval of two [33]. The data obtained was evaluated based on average contig length, N50 values, and percentage of reads assembled (Additional file 2). Assembly at a K-mer value of 51 was found to be optimal for transcriptome assembly of both Heera and Varuna. A total of 189,428 and 190,629 contigs were obtained for Heera and Varuna, respectively.

### Identification of A and B genome specific contigs

The assembled contigs contained both paralogues present within the A and B genomes, and homoeologues across the two genomes. These paralogues and homoeologues were separated in a two-step gene identification process based on the proven premise that paralogues within the A and B genomes are more divergent compared with respective homoeologues between the two genomes [34].

In the first step, the assembled contigs of *B. juncea* lines were searched against protein sequences of *B. rapa* line Chiifu (Brassica_rapa_v1.2.pep; available at the BRAD database) using the BLASTX program. Contigs showing similarity with proteins recorded in the *B. rapa* proteome database at an E-value of ≤ 1e^−05^ were grouped against the listed proteins. Based on this analysis, 31,606 *B. rapa* gene models were found to be represented in the assembled contigs of Heera. A similar approach for Varuna identified 31,335 gene models in the BRAD database in the Varuna transcriptome assembly.

**Figure 1:**
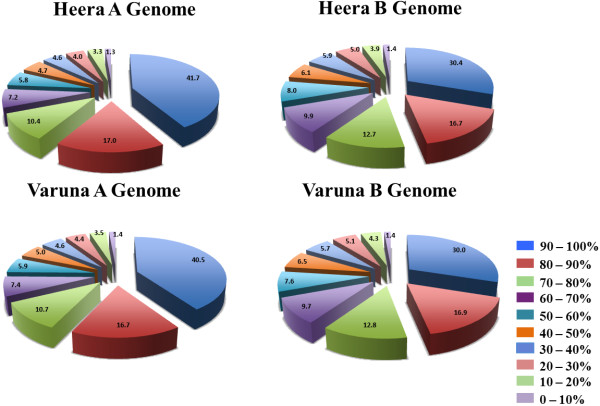
**Coverage of the assembled gene models in the Heera and Varuna genomes**. Assembled gene model coverage in the Heera A, Heera B, Varuna A, and Varuna B genomes compared with gene models described for *B. rapa* in the BRAD database. More than 70% of identified gene models could be assembled at greater than 60% coverage.

In the second step, sequences grouped based on protein similarity were compared at the nucleotide level with *B. rapa* CDS sequences available in the BRAD database (Brassica_rapa_v1.2.cds), and to the transcriptome sequences of two *B. nigra* lines, Sangam and IC2782, that have been analysed in our laboratory (unpublished data). Custom Perl scripts enabled contigs to be grouped based on their maximum identity with either A or B genome reference sequences. Contigs that showed ≤ 80% sequence identity in ≤ 100 bp sequence stretches were removed from the analysis. At this step, the paralogous and homoeologous contigs could be identified and separated. All contigs showing identity to either the *B. rapa* line Chiifu gene model or its homoeologues in *B. nigra*, as per the criteria described above, were considered to be part of the A or B genome gene model. For Heera, 21,212 gene models representing the A genome, and 19,195 representing the B genome were identified. For Varuna, 21,046 and 19,329 gene models could be identified for the A and B genomes, respectively. Gene models were grouped into ‘*B. rapa* vs Heera A genome’, ‘*B. nigra* vs Heera B genome’, ‘*B. rapa* vs Varuna A genome’ and ‘*B. nigra* vs Varuna B genome’; these groups are described in further detail in Additional file 3. Analysis of the read length of the identified gene models for both Heera and Varuna revealed that over 70% of the gene models had > 60% coverage in both A and B genomes (Figure 1). Approximately 40% of the gene models for the A genome had > 90% coverage. Whereas, only 30% of the gene models for the B genome had > 90% coverage (Figure 1).

5,648 contigs of Heera, and 5,559 contigs of Varuna had no similarity to predicted protein sequences reported for *B. rapa*. The contigs were also checked against the *B. rapa* genoeme sequences and contigs showing >80% identity in > 100 bp sequence with the *B. rapa* genome were removed from further analysis. The remaining unique contigs were compared with the *B. nigra* transcriptome sequences. 2,223 contigs with high level of identity with the *B. nigra* transcriptome sequences were identified as B genome specific contigs.

### Identification of SNPs between Heera and Varuna and marker development

**Figure 2:**
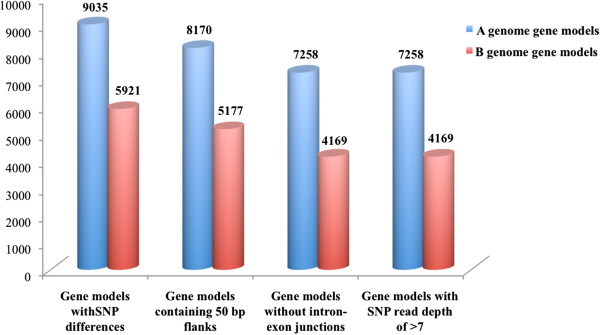
**Number of gene models obtained after each step of marker development**. A larger number of A genome gene models were identified with SNP differences than B genome gene models. At each step of marker development, gene models not meeting the criteria for marker development were removed.

Of the 21,212 and 21,046 A genome specific gene models identified for Heera and Varuna, 17,849 gene models had overlapping sequences. Similarly, of the 19,195 and 19,329 B genome gene models for Heera and Varuna, 16,748 gene models had overlapping sequences. The MUMmer program [35] identified 135,693 SNPs between the allelic gene models of the two lines. These included 85,437 SNPs specific to the A genome, and 50,256 SNPs specific to the B genome. 9,035 gene models from the A genome and 5,921 gene models belonging to the B genome were identified with SNPs (Figure 2). For the A genome 49.3% (8,814 out of 17,849) gene models did not show any nucleotide variations between Varuna and Heera, and for the B genome 64.4% (10,817 out of 16,748) gene models were found to be fully conserved. The frequency of SNPs in the A and B genomes of *B. juncea* revealed that A genomes of Heera and Varuna are more variable and therefore, more diverse than the B genomes of the two lines.

The 14,956 gene models with SNPs (9,035 from A genome; 5,921 from B genome) were classified into three categories i) unique to the B genome (only one copy in *B. juncea*) ii) present as one copy in the A genome and one homoeologue in the B genome, (two copies in *B. juncea*) and iii) more than one paralogue in the A genome and one or more homoeologue(s) in the B genome. Further, SNPs suitable for KASPar technology [36] were identified. This analysis yielded 11,427 SNP containing gene models, 7,258 from the A genome and 4,169 from the B genome (Figure 2).

As comparative analysis of the A and B genomes was one of the major objectives of this study, developing markers for the gene models present as a single copy in the A genome that also had a single copy homoeologue in the B genome, was considered the ideal strategy for genome wide linkage analysis, and for studying the comparative organization of A and B genomes. The positions of A genome gene models were checked in the *B. rapa* database [26, 27] for selecting SNP markers that were well dispersed throughout all 10 A genome chromosomes, and representating all gene blocks. 1,175 markers were identified, and these were classified into three categories: i) those developed from A genome gene models and their homoeologues in the B genome (147 markers from A and 147 markers from B genome) ii) those developed from A genome gene models, but no markers developed from their B genome homoeologues due to lack of polymorphism (465 markers), and iii) those developed from B genome models which had conserved homoeologues in the A genome (269 markers). Additionally, 147 markers were selected from B genome specific gene models to enrich marker coverage of the less explored B genome. The sequences and features of the selected 1,175 SNP markers are provided in Additional file 4.

### SNP marker based mapping in B. juncea

Successful KASPar assays could be designed for 1079 (91.8% success rate) of the 1175 *in silico* selected SNP markers. Of these, 1051 were found to be polymorphic between Varuna and Heera, the parents of the DH mapping population. Fifty-two of these markers showed significant deviations from the expected segregation ratios and were not used for mapping. A linkage map was developed using genotyping data of 999 SNP markers along with 709 IP markers developed earlier [32]. This linkage map, with 1708 loci distributed over 18 LGs (A1 to A10 of A genome; B1 to B8 of B genome) is given in Additional file 5. Block arrangement in relation to *A. thaliana* gene blocks A–X is also provided in the map. The total map length is 1933.5 cm, with an average interval size of 1.4 cM. Constituent A and B genomes showed genetic map lengths of 983.1 and 950.4 cM, with 997 and 711 markers, respectively (Additional file 6). All the A genome specific markers mapped on A genome LGs at the expected positions vis-à-vis BRAD database [26]. The B genome markers mapped to the B genome LGs, thereby showing that the identification of A and B genome contigs, described earlier, is largely accurate.

Genes involved with glucosinolate content had earlier been mapped on LGs A2, A3, A9, and B1 and those involved with erucic acid on LGs A8 and B7 [16, 17, 20, 21]. Introgression of low glucosinolate and ‘0’ erucic acid loci from Heera into Indian types of *B. juncea* with available markers [32] led to a reduction in yield of the recipient lines, most likely due to linkage drag. Therefore, a minimal donor region around these genes needs to be introgressed into the recipient lines to maintain yield potential. The transcriptome data generated in this study was used to analyze mapped loci for the presence of SNP containing genes. Approximately 200 genes, which flanked candidate genes, were selected from the *B. rapa* reference genome [8, 27] and checked for representation in the transcriptome database and for the presence of SNPs in the represented genes. Approximately 50% of gene models were present in the transcriptome database. For glucosinolate loci on LGs A2, A3, and A9 over 30% of gene models around the candidate gene were found to have SNPs, while for the glucosinolate locus on LG B1 25% of gene models had SNPs. For low erucic acid locus A8 and B7, > 50% gene models were represented in the assembled transcriptome, and approximately 24% and 15%, were marked by SNPs in the A and B genomes, respectively. We developed 56 co-dominant markers for genes around those loci; these could be mapped to corresponding locus positions. Information on available genes with SNP differences and the developed markers is provided in Additional file 7.

### Gene block arrangement on the A and B genomes of B. juncea

**Figure 3:**
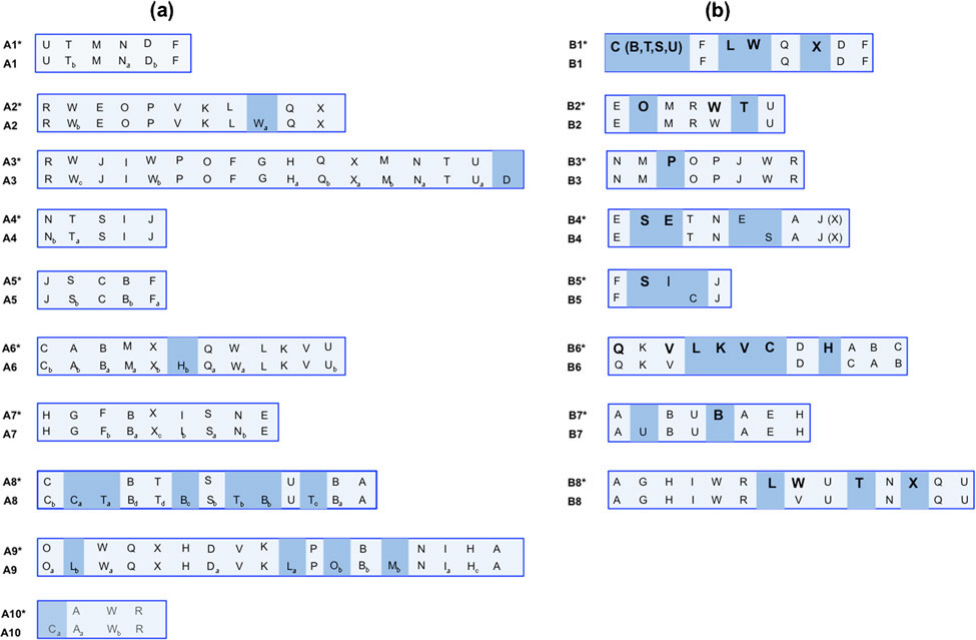
**Comparative block arrangement in the LGs of the A and B genomes of** ***B. juncea*** **. (a)** LGs A1*–A10* (present study); A1–A10 (reference A genome [25]) **(b)** B1*–B8* (present study); B1–B8 (consensus of *B. juncea* B genome map [32] and *B. nigra* map [37]). For the A genome, blocks that could not be identified in relation to the reference genome are highlighted by darker shading. For the B genome, newly identified blocks are shown in bold, and blocks identified in only one of the two maps being compared are highlighted by darker shading.

The genome sequence of *B. rapa* line Chiifu has recently been used [25] to position all 24 triplicated ancestral blocks (A–X) along the 10 LGs. The extent of the gene fractionation of each of these triplicated blocks allowed the following classifications: MF2 (most fractionated), MF1 (medium fractionated), and LF (least fractionated) [8, 24, 25]. We compared the block arrangement on the A genome of *B. juncea* using SNP/IP markers with sequence based arrangements [25] to ascertain the extent of coverage. For *B. juncea* A genome LGs, a block was identified even when a single marker of that block could be mapped to regions where a block had been identified in the *B. rapa* reference genome [25]. Based on this criterion, we could place most of the blocks defined in the reference genome of *B. rapa* onto our map. Gene collinearity and block arrangement patterns were the same in the A genome of *B. juncea* vis-à-vis the A genome of *B. rapa*. Blocks that could not be identified in this study were as follows: a small block Wa on LG A2, block D at the distal end of LG A3, block Hb on LG A6, blocks La, Lb, Ob, and Mb on A9, and block C on A10 (Figure 3a). On LG A8, blocks B, T, and S could not be separated as per the reference genome. Additional markers will help to identify some of these minor blocks.

We identified 67 blocks in the B genome (Figure 3b). Blocks in the B genome were marked only when two or more block specific markers were mapped in that region. As the coverage of the A genome in this study is fairly extensive and accurate, it is likely that the block coverage obtained for the B genome is substantial. A comparison was made with the block arrangement suggested earlier for the B genomes of *B. juncea*[32] and *B. nigra*[37]. A number of new blocks were identified (Figure 3b). On the top end of LG B1, where no blocks were placed in the earlier map [32], block C, and a few gene markers from the B, T, S, and U blocks (constituting around 9% of the mapped area) were identified. Blocks L, W, and X were also identified on LG B1. On LG B2, blocks O and T, which were not discerned earlier, and block W which was previously reported in *B. nigra*, were identified. The arrangement of the blocks on LG B3 remained the same, with the exception of an additional P block. On LG B4, there seemed to be an insertion of S, T, and N blocks within the E block, and the position of the S block was different to that reported for *B. nigra*. In LG B5, blocks S and I were identified. However, block C, which was reported in *B. nigra*[37], was not identified. A number of additional blocks namely L, K, V, C, H have been placed on LG B6, and block B has been identified on LG B7. The presence of block U between A and B blocks on LG B7 reported in *B. nigra* was not confirmed. Block L has been added on LG B8, and blocks T and X have been identified within Block U. Block V, previously identified in the *B. nigra* map [37] and now classified as Wa [8], could also be placed on LG B8 of *B. juncea*.

### Comparison of gene block arrangement and block fragmentation patterns between the A and B genomes of B. juncea

To compare the block arrangements between LGs of the A and B genomes of *B. juncea*, we first identified the homoeologous blocks between the two genomes. This was possible because some SNP markers represented gene models present as a single copy in both A and B genomes. Blocks were only marked as homoeologous if a minimum of two SNP/IP markers from the same block region were placed on both genomes. As SNP markers were developed from gene sequences with gene ids known in both *B. rapa* and *A. thaliana*, the gene content within the identified homoeologous gene blocks could be compared with the block boundaries defined in *A. thaliana*[28] to study block fragmentation patterns between the homoeologous blocks in the A and B genomes.

**Figure 4:**
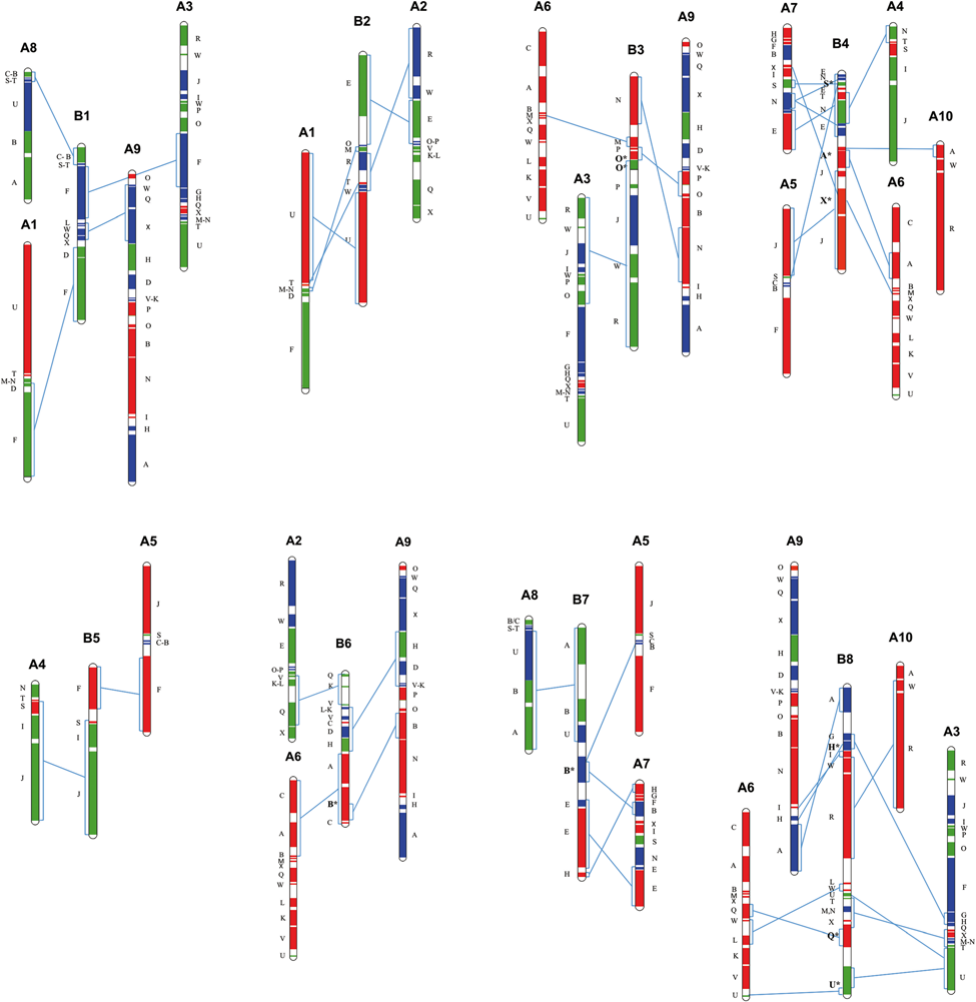
**Block homoeology between the A and B genomes of** ***B. juncea***. Gene blocks on the eight LGs of the B genome (B1-B8) that show homoeology to corresponding blocks on the A genome LGs are shown by the connecting lines. Blocks showing variations in their fragmentation pattern in the B genome are shown in bold with an asterisk. Genomic blocks assigned to the subgenomes LF (red), MF1 (green) and MF2 (blue) are colour coded as in the previous *B. rapa* study [25].

The comparative arrangement of the homoeologous blocks and block fragmentation patterns of all *B. juncea* LGs, along with the markers used in this study, are shown in Figure 4, Additional file 8, and are described below.

LG B1 gene blocks were homoeologous to blocks spread over four A genome LGs, namely A8 (C, B, S, T), A3 (F), A9 (L-W-Q-X), and A1 (D-F). The blocks at the top end of this LG showed gene collinearity with LG A8, but could not be separated into distinct blocks. In the middle section of LG B1, the contiguous block arrangement L-W-Q-X showed homoeology to a similar arrangement on LG A9, with all blocks being from subgenome MF2. No changes were observed in the gene order and orientation of blocks F and D, which showed homoeology to LG A1. The second F block on this LG showed homoeology to the F block of LG A3 belonging to sub-genome MF2.

As with IP markers [32], the gene blocks on LG B2 were homoeologous to LG A2 (R-W-E-O), and A1 (U-T-M). Single markers from block P on LG A2 and from block N on LG A1 also suggested the presence of these blocks on this LG.

The block order N-M-P-O-P-J-W-R on LG B3 showed homoeology with blocks from three A genome LGs: A9 (N, P-O), A6 (M), and A3 (O-P-J-W-R). Of the two P blocks on LG B3, the one next to the M block showed gene collinearity with the P block on LG A9, and the other with that from LG A3. Block O was a mix of gene markers from LGs A9 and A3, with the upper half showing gene collinearity with the LG A9 O_(LF)_ block, and the lower half with the LG A3 O_(MF1)_ block. Within the block order R-W-J-P-O, which shares homoeology with LG A3, one marker each for W and I blocks was located between J and P blocks in LG B3 (Additional file 8), suggesting that these two blocks may also be present on LG B3.

LG B4 was previously shown to be homoeologous to LG A4 along its entire length [32]. In this study it was shown to contain gene blocks homoeologous to five A genome LGs: A4 (N, T), A5 (J, S), A6 (A, X), A7 (E, N, S, X), and A10 (A). This LG is highly chimeric with four of the blocks (A, S, X, N) showing variations in block fragmentation pattern vis-à-vis their homoeologous blocks on the A genome LGs. Block E was observed to be disrupted due to the presence of blocks S, T, and N within this block. Block A_(LF)_ on LG B4, present as a complete block as defined in *A. thaliana* (At1g02220–At1g19330), is present on two different LGs in the A genome: LG A10 (At1g02220–At1g07630) and LG A6 (At1g07640–At1g19330). Similarly block S_(MF1)_, which is disrupted in the A genome to Sa (At5g33210–At5g37810) on LG A5 and Sb (At5g37830–At5g41900) on LG A7, is present as one unit on LG B4. Block X_(LF)_, broken into three fragments in the A genome, Xa (At5g60810–At5g61760) on LG A3, Xb (At5g61770–At5g65925) on LG A6, and Xc (At5g65930–At5g67385) on LG A7 is present as a combined Xb and Xc block on LG B4. Gene markers from block Xa mapped onto LG B8. Block N on LG B4 had gene markers from At3g51870–At3g62790 of subgenome MF1 of LG A4 and At3g52770–AT3G62790 from LG A7 belonging to subgenome MF2 (Additional file 8).

LG B5 had an F block homoeologous to the F block of LG A5. However, blocks J-I-S on this LG had homoeology to a similar block arrangement on LG A4. In our earlier research [32] LGs A5/B5 were shown to be homoeologous along their entire length. However, evidence from the present study showed that LG B5 had homoeologous blocks from two LGs of the A genome.

LG B6, which was previously shown to be homoeologous to LG A6 [32], had homoeology to blocks present on three different LGs, A2 (Q-K-V), A6 (A-B-C), and A9 (L-K-V-C-D-H, B). The block arrangements Q-K-V and L-K-V-C-D-H showed gene collinearity along the entire stretch in the two genomes. The A and B blocks of the arrangement A-B-C on LG B6 showed continuous sequence collinearity with *A. thaliana* genes At1g07640–At1g31750, whereas A and B blocks of similar arrangement in LG A6 of the A genome had block A spanning *A. thaliana* genes At1g07640–At1g19330 and block B from At1g19850–At1g21910. Block C on LG B6 had markers from At1g47960–At1g56120 (Additional file 8), as did LG A6. According to sequence collinearity, the arrangement of these blocks in the B genome was Ab-B-Cb, as compared with Cb-Ab-Ba in the A genome.

LG B7 had homoeology with gene blocks on LGs A5 (B), A7 (B, E, H), and A8 (A-B-U). Block B, in the arrangement A-B-U, showed sequence collinearity with *A. thaliana* genes At1g19850-At1g29020 on LG B7, whereas the homoeologous B_(MF1)_ on LG A8 has been reported to be present as four segments, Ba, Bb, Bc, and Bd, which are disrupted by other blocks (Figure 3a) [25]. In the arrangement A-B-U, only genes from segment Ba are present along with complete A and U blocks on LG A8. Another B block observed on this LG showed homoeology to B_(MF2)_ blocks present on LGs A5 (Bb) and A7 (Ba) in the A genome (Additional file 8). Block E on LG B7, which showed collinearity to the E block on LG A7, is also composed of E blocks belonging to subgenomes LF and MF2, as in the A genome.

LG B8 is highly chimeric, and has the maximum number of blocks. Blocks on this LG show homoeology with blocks of four LGs from the A genome, A9 (A, H, I), A10 (R-W), A6 (L-W, Q, U), and A3 (G-H, T-N-X, U). Three of the blocks show differences in their fragmentation pattern in the A and B genomes. The block arrangements R-W, L-W, and T-N-X from LGs A10, A6, and A3 respectively, showed gene collinearity along their entire stretch in the two genomes. The block arrangement G-H-I on LG B8 had gene markers from three A genome LGs: block G_(MF2)_ from A3; block H_(MF2)_ from A3(Ha), A6(Hb), and A9(Hc); and block I_(LF)_ from A9. The gene order in this block arrangement in the B genome showed continuous sequence collinearity with *A. thaliana* genes At2g05170–At2g26660s. Block Q on LG B8 had markers present from *A. thaliana* genes AT5g23030–AT5g28470, which were also present as Qa on LG A6 and Qb on LG A3; both belonging to subgenome LF. Similarly, block U on LG B8 had gene markers from *A. thaliana* genes At4g16250–At4g38770, which were present as Ua on LG A3 and Ub on LG A6 in the A genome. The U block on B8 was observed to be disrupted differently due to the presence of blocks T, N, X, and Q from LG A3 within this block, with *A. thaliana* orthologues At4g16250–At4g24180 forming part a, and At4g24190–At4g38770 part b.

## Discussion

There were two major objectives for this study on RNA-seq for SNP discovery in *B. juncea* – an assessment of the availability of SNP markers for general mapping and specific -region fine mapping, and analysis of comparative genome organization between the constituent A and B genomes. The frequency of SNPs in the lines Heera and Varuna revealed that the A genome of the two lines is more diverse than the B genome. In our earlier *B. rapa* RNA-seq study we found that there was a very high SNP frequency between *B. rapa* vegetable and oleiferous types, and between oleiferous lines of ssp. *trilocularis* and ssp. *oleifera*[22]. Low variability was found only between two *B. rapa* ssp. trilocularis types YSPB24 and tetralocular, both of which are yellow sarson types. Our results demonstrate that only a limited variability of *B. rapa* types have been captured in the allopolyploid *B. juncea* lines of the two gene pools (Additional file 9).

The available Heera and Varuna SNPs allow both general and fine-mapping to be successfully performed using SNP markers. It was possible to carry out fine-mapping of regions containing the high value traits of low erucic and low glucosinolate in the Heera line. We made use of co-dominant SNP markers marking both SNPs and HSVs. However, many more of the genes in the region can be covered by gene-specific dominant markers (Additional file 7). This information will help in converting major Indian gene pool lines into ‘Canola’ quality or ‘00’ lines through marker based introgressions.

As RNA-seq based markers are gene specific, the limited number of SNP markers developed in this study used in conjunction with gene based IP markers provided new insights into the arrangement of homoeologous blocks on the LGs of A and B genomes of *B. juncea*. The U’s triangle species have gone through two events of polyploidization – ***b*** and ***U***[23, 38]. The ***b*** event gave rise to the three diploids – *B. rapa* (AA), *B. nigra* (BB), and *B. oleracea* (CC). The much more recent ***U*** event led to the development of the three allotetraploids – *B. juncea* (AABB), *A. napus* (AACC), and *B. carinata* (BBCC). The ***U*** event is characterised by suppression of pairing between homoeologous chromosomes [39, 40], and a maintaining of the gene block arrangement of the parental diploid species. Evolution of the three diploid genomes A, B, and C is much more complicated. These diploid species are derivatives of a genome triplication event of an n = 7 karyotype ancestor [25]. This is strongly supported by triplication of each gene block (A-X) identified in the ancestral crucifer karyotype (n = 8) or proto-calepineae karyotype (n = 7) [25] in the A, B, and C genomes. Genome triplication has been observed in many other taxa of tribe Brassiceae by *in situ* hybridization [41] and genetic mapping [42–44].

The following evolutionary aspects are well established for Brassica species

- *B. rapa* sequencing data has shown that A–X blocks of *A. thaliana* are present in triplicate in the A genome. Based on gene fractionation, the three genomes that exist today in *B. rapa* have been classified as MF2, MF1, and LF [8, 24]. These are represented as X, Y, and Z progenitor genomes, respectively.
- It has been suggested that the ***b*** event hexaploidization occurred in two steps – first the formation of a tetraploid (MF1 × MF2; or X × Y) followed by the entry of the Z genome, which is the least fractionated in *B. rapa* in terms of gene loss [8, 24].
- Paralogous genes of X, Y, and Z genomes that contribute to the A genome, and X′, Y′, and Z′ genomes of the B genome are more divergent than homoeologous genes across X-X′, Y-Y′, and Z-Z′ [32]. Synonymous substitution rates between X, Y, and Z paralogues show an evolutionary divergence time of ~12 Mya, and between the X-X′, Y-Y′, and Z-Z′ homoeologues, a divergence time of ~7 Mya. The X, Y, and Z progenitor genomes (species) evolved from a *Brassica* ancestor, which split from *A. thaliana* approximately 15 Mya [34, 45, 46].
- Very strong evidence for a minimum of two reciprocal crosses for the ***b*** event of polyploidization comes from the presence of two distinct plastid lineages in the tribe *Brassiceae* – Rapa/Oleracea (A and C genome) lineage and Nigra/Sinapis (B genome) lineage [47–50]. The plastid genome of the two lineages diverged ~12 Mya, a time scale similar to the divergence of the X, Y, and Z genomes that constitute the genome of present day diploid species of the U’s triangle. Phylogenetic analysis of 89 *Brassica* species, based on plastid genome intergenic region sequences, revealed eight plastid lineages in the tribe *Brassicaceae*[51]. Therefore, the ***b*** event could have involved multiple crosses between divergent progenitor genomes.

We looked at the A and B nuclear genome organization to answer the following questions:-

- Were A and B genomes independent hexaploidization events, or were they derived from a single hexaploidization?
- Did chromosome rearrangements take place in the XXYY tetraploid, and again when the Z genome entered to complete the ***b*** event? Or was XXYY a strict allotetraploid, and chromosome changes occurred only after crosses with Z genome species?

**Figure 5:**
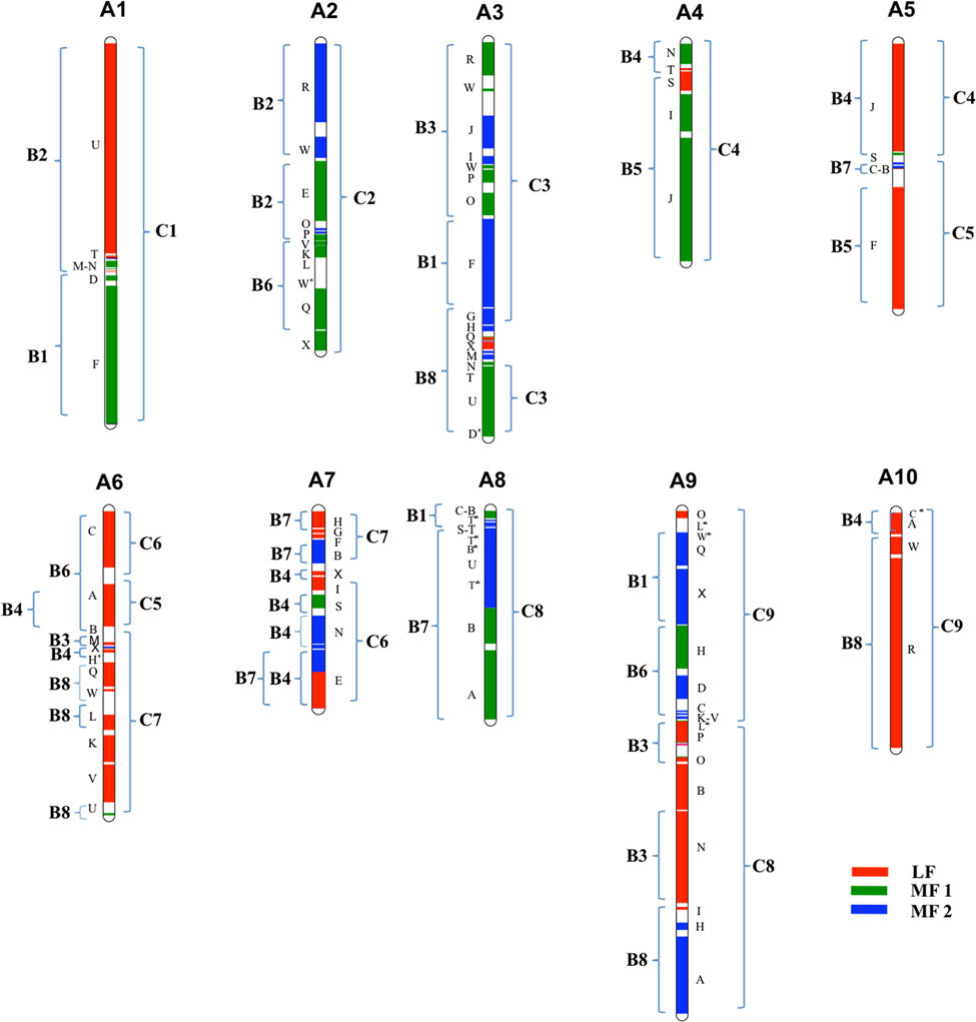
**Chromosome collinearity comparison among A, B, and C genomes**. A and C genome collinearity is based on a *B. napus* study [52]. LF (red), MF1 (green), and MF2 (blue) blocks are colour coded as in the previous *B. rapa* study [25]. The figure shows that the block arrangement pattern between the A and B genomes is much more complex than that between the A and C genomes.

Comparison of collinearity between the A, B, and C genomes clearly show that while simple translocations and inversions could explain the divergence of A and C genome [52], the same cannot be claimed for B genome (Figure 5). With the exception of LGs B2 and B5, which can be derived from two LGs of the A genome, gene block arrangements on all other LGs of the B genome show blocks homoeologous to blocks from three or more LGs of the A genome (Figures 4, 5). In addition to the variations observed in the genomic block architecture, analysis of gene collinearity within the gene blocks of the A and B genomes vis-à-vis *A. thaliana* showed variations in block fragmentation patterns between the two genomes. Some gene blocks, like A_(LF)_, B_(LF, MF2)_, H_(MF2)_, Q_(LF)_, S_(MF1)_, U_(MF1)_, and X_(LF)_ are disrupted into two or more sub-blocks in the A genome, but present as complete blocks in the B genome (Additional file 8). Similarly, genes from blocks such as B_(MF1)_, F_(MF1)_, Q_(MF1)_, T_(MF2)_, and U_(MF2)_ are represented on two different LGs in the B genome, but present as complete blocks in the A genome. Four gene block associations resulting from recombination between homoeologous segments have been reported in the A genome [25], of these only E_(LF)_/E_(MF2)_ could be located in the B genome. Two block associations unique to the B genome were identified, O_(LF)_/O_(MF1)_ on LG B3 and N_(MF1)_/N_(MF2)_ on LG B4.

The presence of two copies of the block association E_(LF)_/E_(MF2)_ (LG A7) on LGs B4 and B7 (Additional file 8) appears to be a unique feature of the B genome, and is not observed in either the A or C genomes. Some parts of blocks such as A_(LF)_, N_(MF2)_, and U_(MF2)_ were duplicated on two different LGs in the B genome (Additional file 8). Another uniqueness of the B genome is the presence of the block arrangement G-H-I on LG B8; this showed continuous sequence collinearity with a similar arrangement in *A. thaliana*, whereas the H block of the similar arrangement in the A genome is fragmented into three sub-blocks, which are present on different LGs, with blocks G and H belonging to subgenome MF2 and block I to subgenome LF (Figure 4). Similarly, blocks A and B of block arrangement A-B-C on LG B6 showed continuous sequence collinearity with *A. thaliana*. The presence of such contiguous arrangements in the B genome is likely to be due to their presence in one of the subgenomes that took part in the hexaploidization event.

The comparison of A and B genomes in this study does not completely rule out the two genomes being outcomes of independent meiotic events from a common hexaploid ancestor. However, the presence of extensive differences in the nuclear genome organization of A and B genomes, coupled with the well-established fact that the two genomes belong to two distinct plastid lineages, makes the hypothesis of A and B genomes being outcomes of independent hexaploidization events the most plausible [53].

Despite a large number of unique block associations in the B genome, some block associations, including R-W-E-O-P (LGs A2/B2), R-W-J-I-P-O (LGs A3/B3), L-K-V-D-H (LGs A9/B6), and A-B-U (LGs A8/B7) are conserved in A, B, and C genomes (Figure 4). Of the common block motifs shared by all three genomes, R-W-E-O-P, R-W-J-I-P-O and A-B-U have also been observed in the related species *Raphanus sativus* and *Sinapis alba*[42–44]. Since the Rapa/Oleracea lineage also includes *R. sativus*, and the Nigra lineage includes *S. alba*, the presence of common motifs in both lineages indicates that these block arrangements took place before reciprocal crosses led to the evolution of the two plastid lineages. Additionally, we observed that all blocks in these motifs belong to subgenomes MF1 and MF2 in all three genomes, indicating that these arrangements had possibly occurred in the ancestral tetraploid (XXYY), which provided MF1 and MF2 genomes for the A and B genomes.

Thus the most plausible hypothesis is that A and B genomes have evolved from independent hexaploidization events [53], and that the tetraploid (XXYY) underwent chromosomal reshuffling and some gene fractionation before hybridization with the Z genome, which is the least fractionated sub-genome present in the A and B genomes.

## Conclusion

RNA-seq of the *B. juncea* lines Varuna (Indian gene pool) and Heera (east European gene pool) provided a large number of SNPs for general and specific -region fine mapping. Large numbers of SNPs were identified between the A and B genomes, and the A genome was found to be more divergent than the B genome of *B. juncea*. A linkage map, developed using SNP and IP markers, identified many new gene blocks in the B genome. A number of genes were marked with co-dominant SNP markers around loci controlling low glucosinolate (LGs A2, A3, A9, and B1) and low erucic acid (LGs A8, B7) traits. This will allow the future transfer of ‘00’ traits from Heera into Indian lines. Comparative analysis of gene block arrangement and genome fragmentation patterns revealed that the constituent A and B genomes of *B. juncea* are diverse, and supported the hypothesis that the two genomes evolved from independent hexaploidization events. Genic SNP markers will help development of physical maps for *B. juncea* and *B. nigra*, and in assembling the *B. nigra* genome.

## Methods

### Plant material, RNA isolation, and transcriptome assembly

Two lines of *Brassica juncea*, namely Varuna (Indian gene pool) and Heera (east European gene pool) were maintained in the field by selfing for more than ten generations. Seedling tissue and plant parts were collected from field grown plants. The following three tissue amalgams were taken and processed for RNA isolation:

i. Five days old seedlings grown in a growth chamber (10/14 h day/night at 20/18°C).
ii. Young inflorescence, comprising unopened flower buds and small leaves from field grown plants.
iii. Post fertilization, 10, 20, 30, and 40 day old pods, all taken together as one sample.

RNA was isolated from each of the three samples types using a Total RNA Spectrum kit (Sigma-Aldrich, St Louis, MO, USA) and subsequently treated with DNase A (Ambion, TX, USA). Equal amounts of RNA, 6 μg from each sample, were pooled and used for cDNA library preparation following the method described for RNA-seq of *B. rapa* lines [22].

Libraries prepared for Varuna and Heera were sequenced as 2×101 nt paired end reads on a Genome Analyzer IIx instrument (Illumina Inc, San Diego, USA). Sequence data were obtained from four lanes of the flowcell for Heera, and from five lanes for Varuna. Quality checking and *de novo* assembly was carried out using the Fastx-toolkit [54] and Velvet *de novo* assembly programs respectively, following the parameters described by Paritosh et al. [22].

### SNP genotyping and construction of linkage map

Oligos for SNP genotyping were synthesized by KASPar technology [36] using FRET quencher oligos competitor allele specific arrays. SNPs between contigs of the two lines were identified using the MUMmer program [35]. SNPs were only selected with read depths of ≥ 7, and were surrounded by a conserved flanking region of ≥ 50 bp on each side. The 101 bp regions containing the variable base were screened for the presence of exon-intron junctions, and any sequences with such junctions removed. This strategy for the development of markers using KASPar technology was earlier used for *B. rapa*[22].

Genotyping of the designed SNP markers was carried out on a F_1_DH (doubled haploid) population of 123 individuals derived from a Varuna x Heera cross. The linkage map was developed as previously described by Panjabi et al. [32]. Linkage groups were established with a minimum LOD threshold of 4.0 using Joinmap 4.0 [55], and the recombination fractions were transformed into map distances with the Kosambi function [56]. Linkage groups were drawn using Mapchart 2.2 [57].

### Availability of supporting data

The raw reads data (SRA) and transcriptome shortgun assembly data (TSA) have been submitted under the NCBI BioProject ID: PRJNA245462 (http://www.ncbi.nlm.nih.gov/bioproject/?term=PRJNA245462). Information about the samples used in the transcriptome sequencing of *Brassica juncea* lines Heera and Varuna has been deposited with NCBI BioSample accession numbers SAMN02738248 and SAMN02741811, respectively.

## Electronic supplementary material

Additional file 1: **Sequencing and assembly statistics of** ***B. juncea*** **lines Heera and Varuna**. (DOCX 14 KB)

Additional file 2: **Comparison of total contig number, average contig length, and N50-length obtained after Velvet assembly**. Figures A and B represent the contig assembly results of *B. juncea* line Heera and Varuna, respectively. The bars indicate total number of contigs assembled (primary axis). The green line represents N50 contig length, while the red line indicates average contig length (secondary axis). (TIFF 242 KB)

Additional file 3: **Grouping of A or B genome specific contigs of** ***B. juncea*** **with their homologs in** ***B. rapa*** **cds (BRAD database) and** ***B. nigra*** **transcriptome (unpublished data)**. The homologs are represented as ‘*B. rapa* vs Heera A genome’, ‘*B. nigra* vs Heera B genome’, ‘*B. rapa* vs Varuna A genome’ and ‘*B. nigra* vs Varuna B genome’ databases. In all four databases column A represents either *B. rapa* gene models (Bra……) or their corresponding homologues in *B. nigra* (Bni……); column B onwards show contigs obtained from transcriptome sequencing of *B. juncea* lines. (XLSX 11 MB)

Additional file 4: **Description of sequences used to identify SNPs**. The data contains the description of the genes for which the SNP based markers were developed. Column A – marker id; column B – *A. thaliana* homolog id; column C – block position of the gene model; column D – chromosomal position of the *B. rapa* gene model; column E – gene id in *B. rapa* or in *B. nigra*; column F – genome position of the gene model; column G – chromosomal position of the marker after mapping; and column H – sequence used for SNP marker development. Variable bases in the sequences are shown in a bracket. PSVs and HSVs are shown in lower case, and wherever required degeneracy was put in lower case in the marker sequences. *A. thaliana* ids, and corresponding *B. rapa* genes ids were obtained from the BRAD database. (XLSX 161 KB)

Additional file 5: **Linkage map of** ***B. juncea*** **(Varuna x Heera) developed from an F1-DH population using SNP and IP markers**. There are 999 SNP and 709 IP markers present on the linkage map. Linkage groups are named A1–A10 and B1–B8, following the guidelines of an earlier study [32]. Markers are shown on the right of the linkage group bar, and marker positions (cM) on the left. Block positions of the markers are shown on the left of the linkage group bar. Blocks A–X have been given eight distinct colour codes, as per the blocks on eight chromosomes of the progenitor Ancestral Crucifer Karyotype (ACK) genome [25, 52]. SNP markers have the prefix BJ_VH_, and IP markers bear the name of the *A. thaliana* gene id from which they were developed. (PDF 476 KB)

Additional file 6: **Characteristics of the linkage map of** ***Brassica juncea*** **constructed using SNP and IP markers**. (DOCX 17 KB)

Additional file 7: **Fine mapping of low erucic acid and low glucosinolate gene loci**. Gene models containing SNPs around loci encoding erucic acid and glucosinolate traits are shown. Approximately 100 genes around the candidate genes were selected from four glucosinolate loci on LGs A2, A3, A9, and B1 and two erucic acid loci on LGs A8 and B7. Each of these six regions are arranged as ‘A genome gene model’, ‘corresponding *A. thaliana* gene ids’, ‘block position’, and ‘corresponding B genome gene models’. Candidate genes are shown in red, gene models represented in the transcriptome sequencing are shown in green, gene models with SNP differences are shown in orange, and gene models from which markers have been developed are highlighted in blue. (XLSX 36 KB)

Additional file 8: **Block fragmentation in** ***B. rapa*** **and** ***B. nigra***. Number of genomic blocks with their fragmentation patterns in *B. nigra* in relation to *B. rapa*. This table is modified from an earlier study [25]. The positions of different gene blocks on B1–B8 LGs of the B genome of *B. juncea* are marked by bracketed lines on the original table. Bracketed lines show gene ids where markers could be placed on the B genome LGs. Markers were not developed for gene ids shown without bracketed lines. Red highlight: gene blocks without rearrangements; yellow highlight: broken gene blocks; blue highlight: gene blocks with deletions in some of the genes. Some blocks could not be placed on the B genome. (DOCX 105 KB)

Additional file 9: **Comparison of the number of SNPs identified in different lines of** ***B. rapa*** **and in the A and B genomes of** ***B. juncea***. The number of SNPs identified between various lines of *B. rapa* are taken from our earlier study [22]. *B. juncea* data were generated in this study. (DOCX 14 KB)
