# Supplementary material for: RNA-seq based SNPs for mapping in Brassica juncea (AABB): synteny analysis between the two constituent genomes A (from B. rapa) and B (from B. nigra) shows highly divergent gene block arrangement and unique block fragmentation patterns
Source: BMC Genomics. 2014 May 23;15(1):396. doi: 10.1186/1471-2164-15-396 (PMC4045973; doi:10.1186/1471-2164-15-396)
Supplement: Supplementary file 1 — Additional file 1: Sequencing and assembly statistics of B. juncea lines Heera and Varuna. (DOCX 14 KB) [file 12864_2013_6090_MOESM1_ESM.docx]

**Additional Table 1** Sequencing and assembly statistics of Heera and Varuna lines of *B. juncea*

|  | Heera | Varuna |
| --- | --- | --- |
| Total number of reads | 134,372,556 | 172,720,474 |
| Paired ends (after filtering low quality reads) | 128,745,616 | 134,116,723 |
| Number of contigs | 189,428 | 190,629 |
| Percentage of reads assembled | 63.9 | 64.3 |
| Maximum length of contigs (bp) | 11,027 | 8,976 |
| N50 contig length (bp) | 387 | 376 |
| Mean depth of the contigs | 43.44 | 53.18 |
