# Supplementary figures and images for: RNA-seq based SNPs for mapping in Brassica juncea (AABB): synteny analysis between the two constituent genomes A (from B. rapa) and B (from B. nigra) shows highly divergent gene block arrangement and unique block fragmentation patterns
Source: BMC Genomics. 2014 May 23;15(1):396. doi: 10.1186/1471-2164-15-396 (PMC4045973; doi:10.1186/1471-2164-15-396)

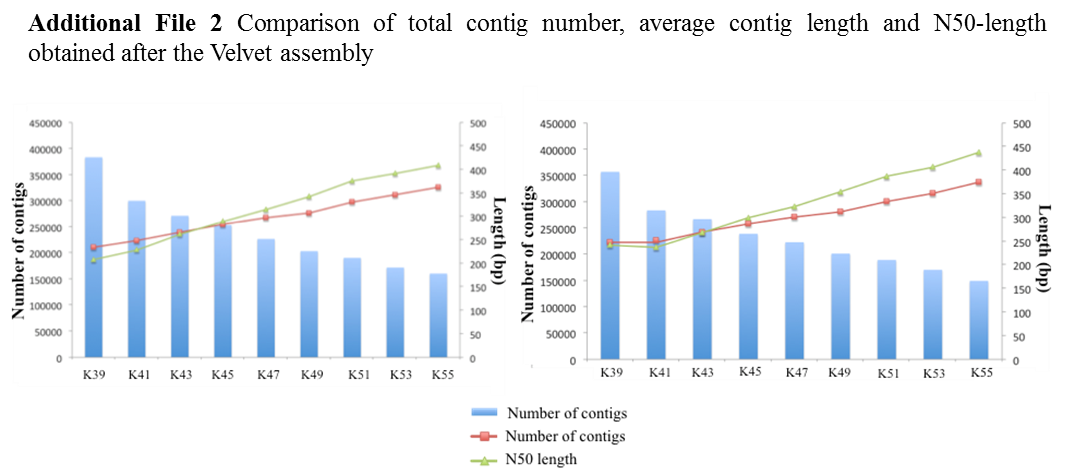

Supplement: Supplementary file 2 — Additional file 2: Comparison of total contig number, average contig length, and N50-length obtained after Velvet assembly. Figures A and B represent the contig assembly results of B. juncea line Heera and Varuna, respectively. The bars indicate total number of contigs assembled (primary axis). The green line represents N50 contig length, while the red line indicates average contig length (secondary axis). (TIFF 242 KB) [file 12864_2013_6090_MOESM2_ESM.tiff]

A1

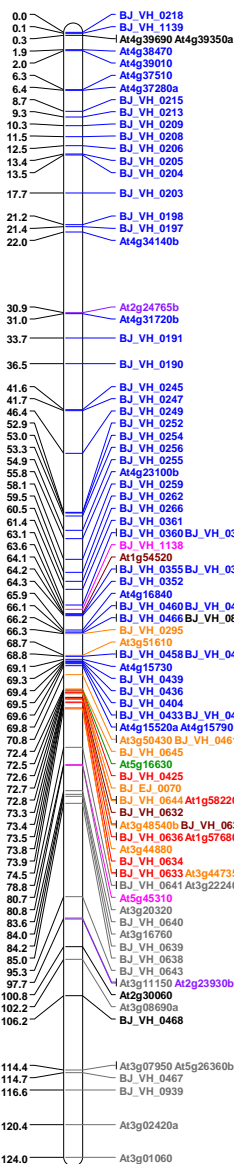

A2

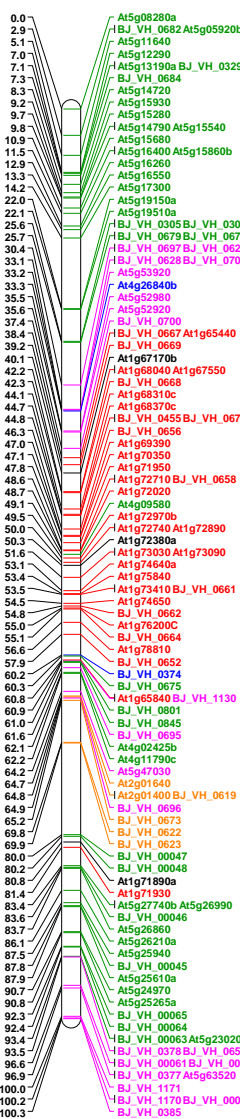

A3

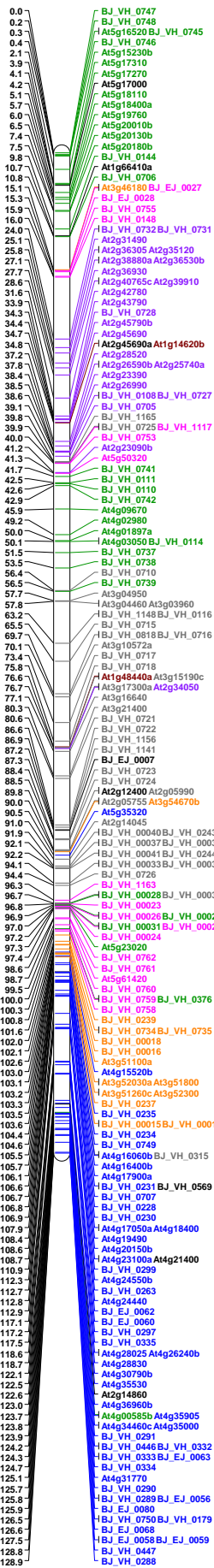

A4

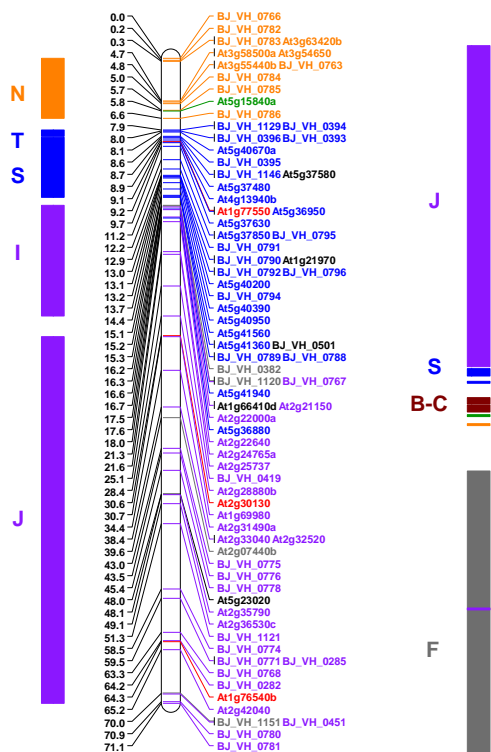

A5

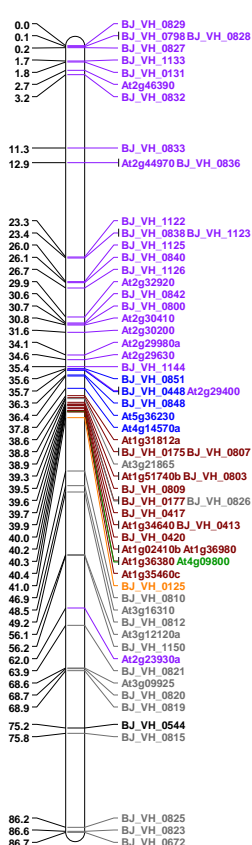

A6

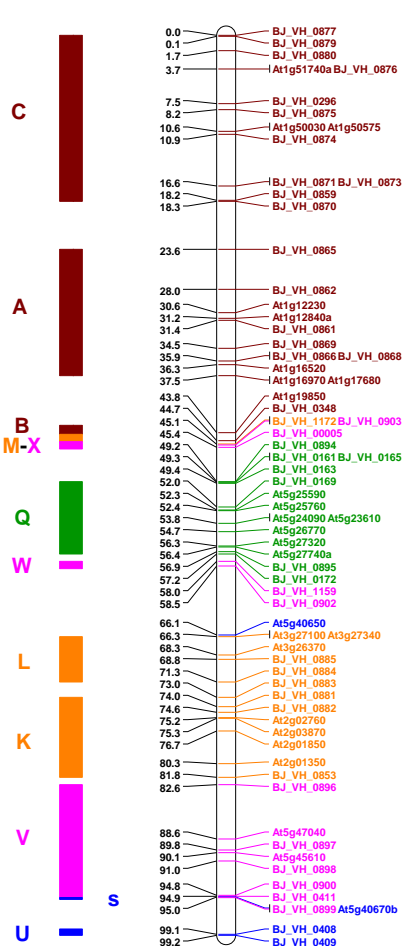

A7

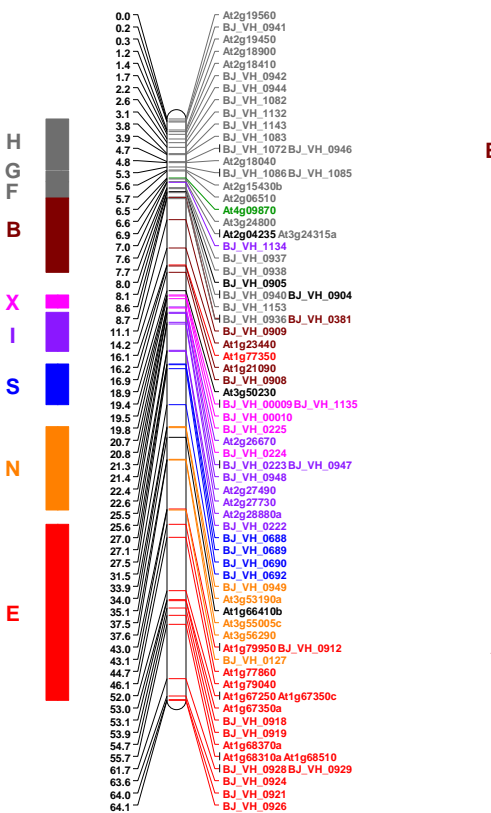

A8

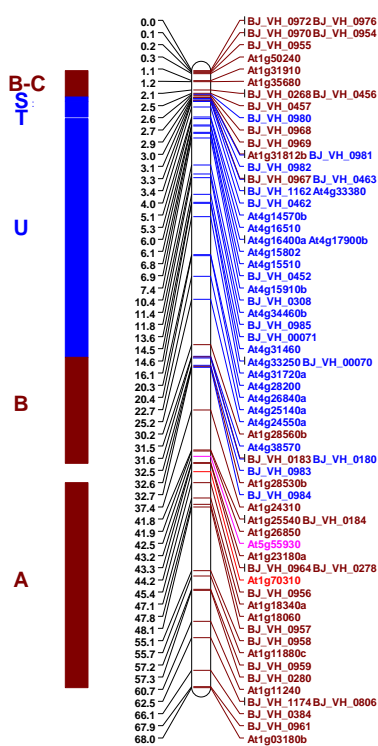

A9

O

Q

X

H

D

V-K

P

V

P

B

N

I

H

A

g

n

r

m

c

k

o

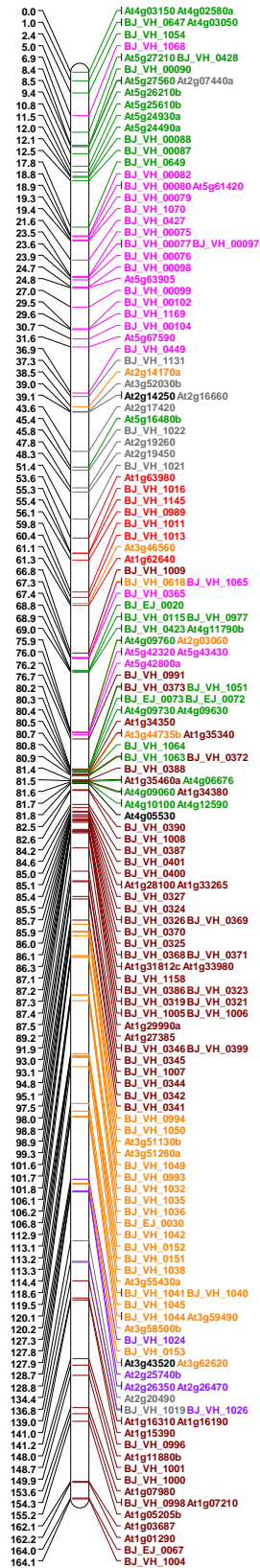

A10

A

W

R

e

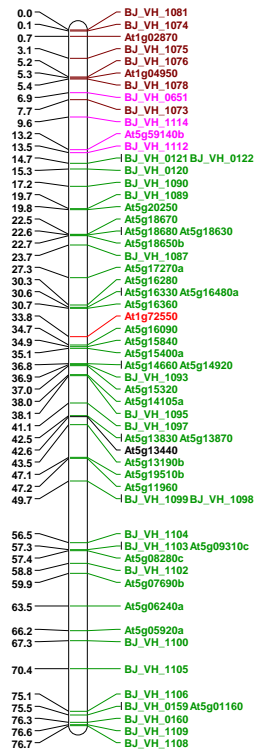

# B1

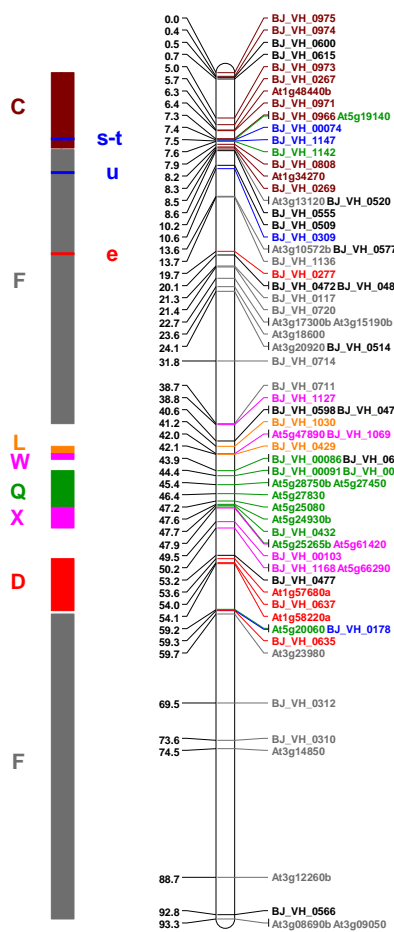

**B2**

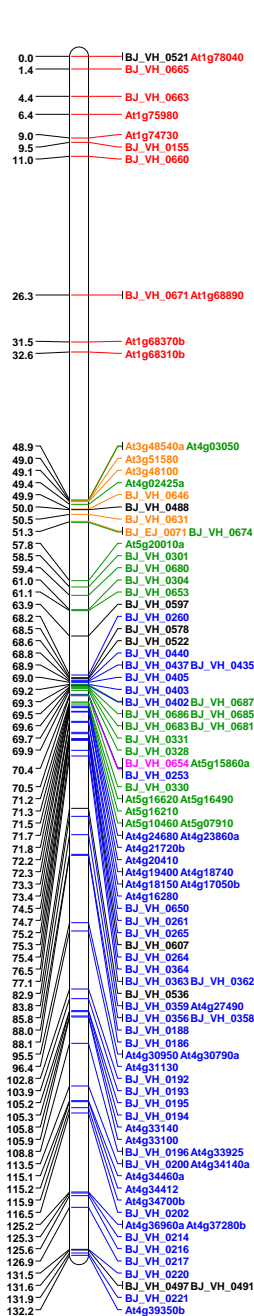

**B3**

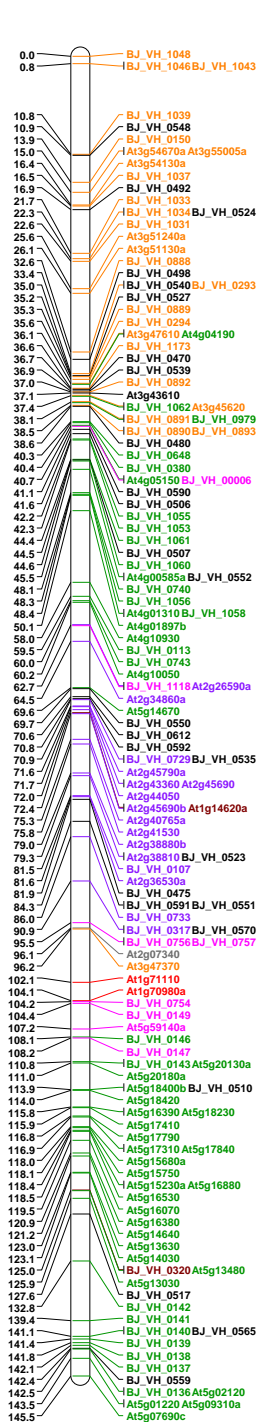

B4

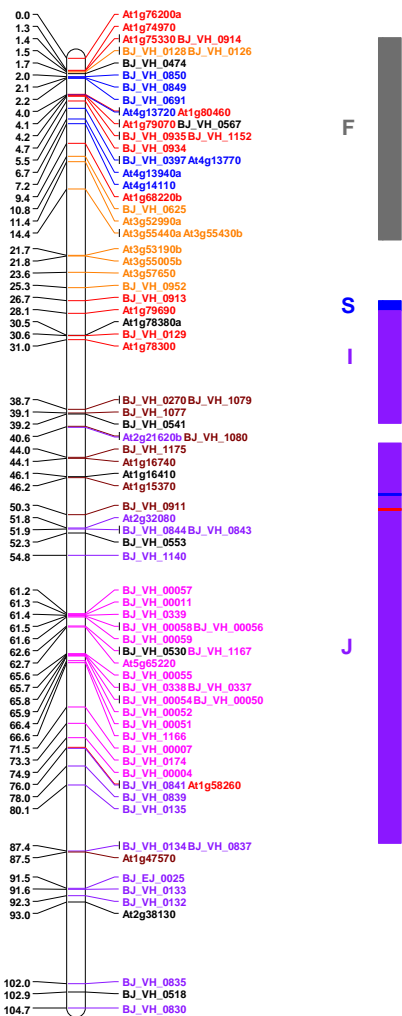

B5

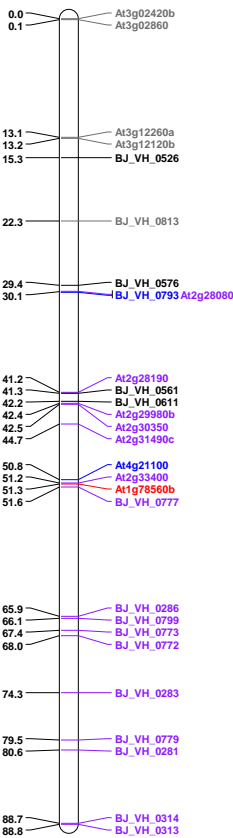

B6

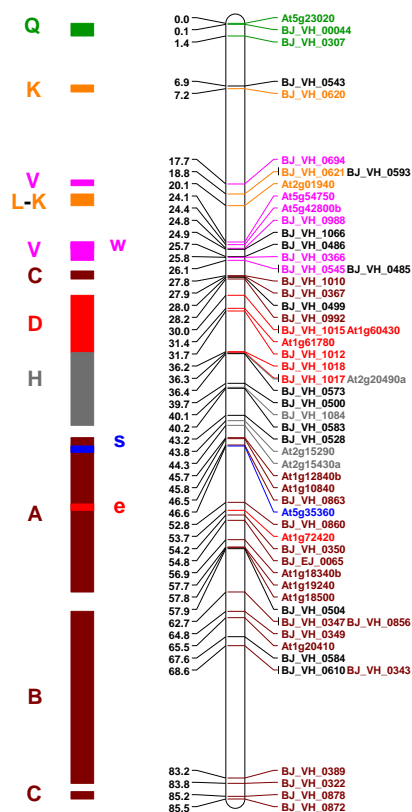

B7

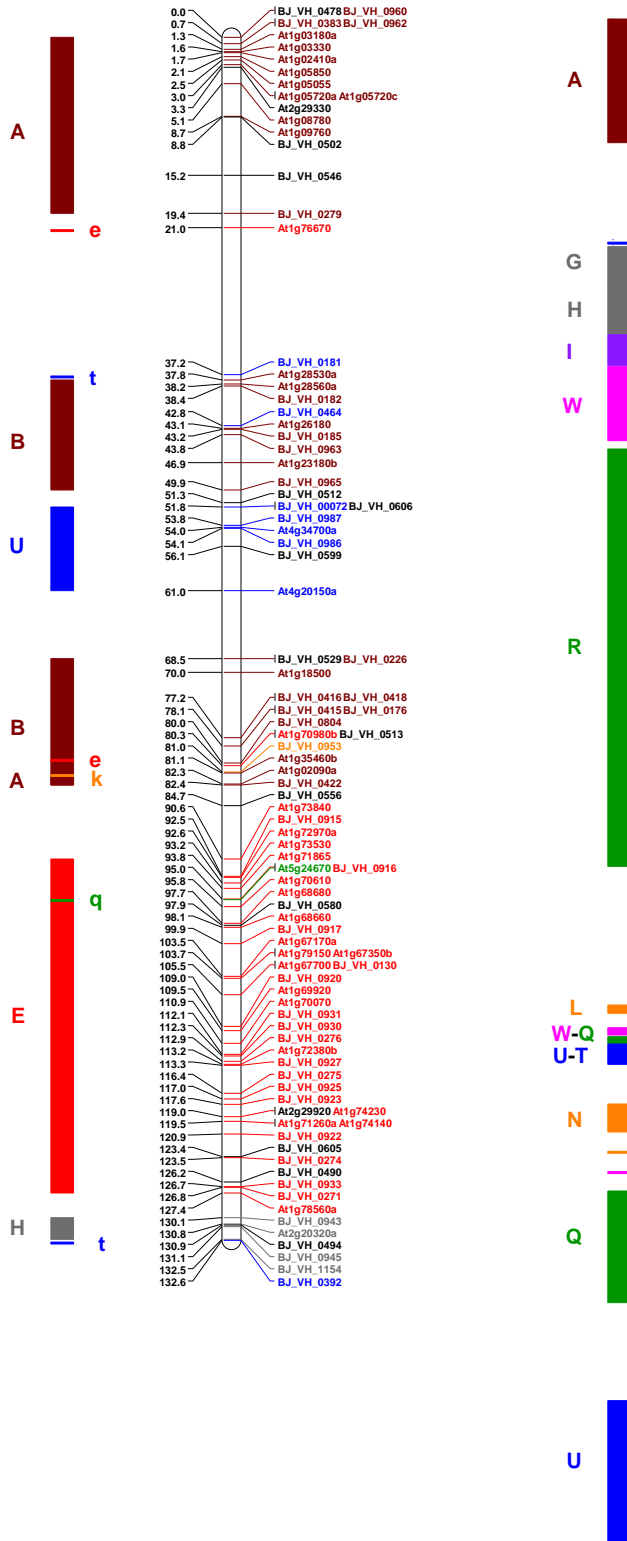

B8

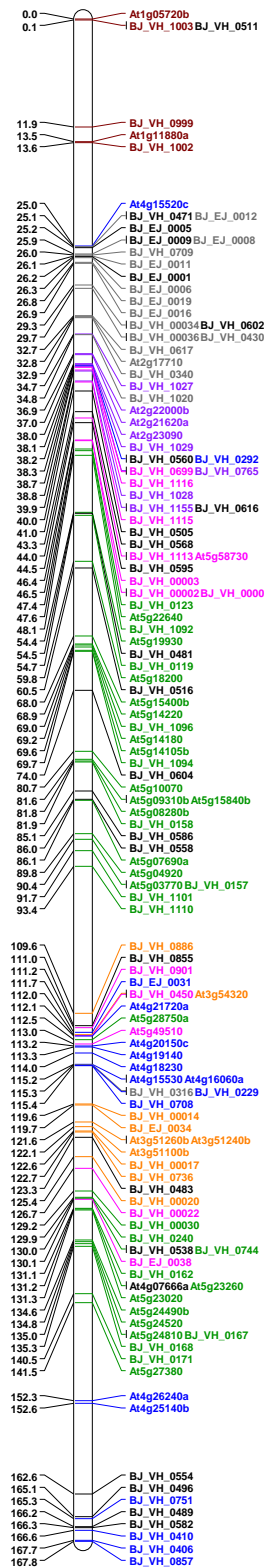

Supplement: Supplementary file 5 — Additional file 5: Linkage map of B. juncea (Varuna x Heera) developed from an F1-DH population using SNP and IP markers. There are 999 SNP and 709 IP markers present on the linkage map. Linkage groups are named A1–A10 and B1–B8, following the guidelines of an earlier study [32]. Markers are shown on the right of the linkage group bar, and marker positions (cM) on the left. Block positions of the markers are shown on the left of the linkage group bar. Blocks A–X have been given eight distinct colour codes, as per the blocks on eight chromosomes of the progenitor Ancestral Crucifer Karyotype (ACK) genome [25, 52]. SNP markers have the prefix BJ_VH_, and IP markers bear the name of the A. thaliana gene id from which they were developed. (PDF 476 KB) [file 12864_2013_6090_MOESM5_ESM.pdf]
