# Supplementary material for: RNA-seq based SNPs for mapping in Brassica juncea (AABB): synteny analysis between the two constituent genomes A (from B. rapa) and B (from B. nigra) shows highly divergent gene block arrangement and unique block fragmentation patterns
Source: BMC Genomics. 2014 May 23;15(1):396. doi: 10.1186/1471-2164-15-396 (PMC4045973; doi:10.1186/1471-2164-15-396)
Supplement: Supplementary file 6 — Additional file 6: Characteristics of the linkage map of Brassica juncea constructed using SNP and IP markers. (DOCX 17 KB) [file 12864_2013_6090_MOESM6_ESM.docx]

| **Linkage group** | **Length (cM)** | **Number of IP markers** | **Number of SNPs** | **Total number of markers** | **Number of intervals** | **Average interval size (cM)** | **Marker density^b^** |
| --- | --- | --- | --- | --- | --- | --- | --- |
| **A1** | 124.0 | 35 | 61 | 96 | 79 | 1.6 | 0.8 |
| **A2** | 100.3 | 64 | 50 | 114 | 90 | 1.1 | 1.1 |
| **A3** | 128.9 | 85 | 107 | 192 | 149 | 0.9 | 1.5 |
| **A4** | 71.1 | 39 | 39 | 78 | 60 | 1.2 | 1.1 |
| **A5** | 86.7 | 23 | 42 | 65 | 54 | 1.6 | 0.7 |
| **A6** | 99.2 | 27 | 44 | 71 | 60 | 1.7 | 0.7 |
| **A7** | 64.1 | 32 | 45 | 77 | 65 | 1.0 | 1.2 |
| **A8** | 68.0 | 34 | 38 | 72 | 59 | 1.2 | 1.1 |
| **A9** | 164.1 | 68 | 101 | 169 | 133 | 1.2 | 1.0 |
| **A10** | 76.7 | 33 | 30 | 63 | 54 | 1.4 | 0.8 |
| **Total** | **983.1** | **440** | **557** | **997** | **803** | **1.2^a^** | **1.0^a^** |
|  |  |  |  |  |  |  |  |
| **B1** | 93.3 | 26 | 47 | 73 | 57 | 1.6 | 0.8 |
| **B2** | 132.2 | 41 | 63 | 104 | 80 | 1.7 | 0.8 |
| **B3** | 145.5 | 60 | 78 | 138 | 109 | 1.3 | 0.9 |
| **B4** | 104.7 | 28 | 57 | 85 | 68 | 1.5 | 0.8 |
| **B5** | 88.8 | 12 | 16 | 28 | 26 | 3.4 | 0.3 |
| **B6** | 85.5 | 17 | 41 | 58 | 51 | 1.7 | 0.7 |
| **B7** | 132.6 | 42 | 54 | 96 | 82 | 1.6 | 0.7 |
| **B8** | 167.8 | 43 | 86 | 129 | 109 | 1.5 | 0.8 |
| **Total** | **950.4** | **269** | **442** | **711** | **582** | **1.6^a^** | **0.7^a^** |
|  |  |  |  |  |  |  |  |
| **Total (A + B)** | **1933.5** | **709** | **999** | **1708** | **1385** | **1.4^a^** | **0.9^a^** |

**Additional file 6** Characteristics of linkage map of *Brassica juncea* constructed with Intron Polymorphism (IP) and Single Nucleotide Polymorphism (SNP) markers

**^a^**Mean values

**^b^**Marker density is defined as the number of markers per centiMorgan
