# Supplementary material for: RNA-seq based SNPs for mapping in Brassica juncea (AABB): synteny analysis between the two constituent genomes A (from B. rapa) and B (from B. nigra) shows highly divergent gene block arrangement and unique block fragmentation patterns
Source: BMC Genomics. 2014 May 23;15(1):396. doi: 10.1186/1471-2164-15-396 (PMC4045973; doi:10.1186/1471-2164-15-396)
Supplement: Supplementary file 8 — Additional file 8: Block fragmentation in B. rapa and B. nigra. Number of genomic blocks with their fragmentation patterns in B. nigra in relation to B. rapa. This table is modified from an earlier study [25]. The positions of different gene blocks on B1–B8 LGs of the B genome of B. juncea are marked by bracketed lines on the original table. Bracketed lines show gene ids where markers could be placed on the B genome LGs. Markers were not developed for gene ids shown without bracketed lines. Red highlight: gene blocks without rearrangements; yellow highlight: broken gene blocks; blue highlight: gene blocks with deletions in some of the genes. Some blocks could not be placed on the B genome. (DOCX 105 KB) [file 12864_2013_6090_MOESM8_ESM.docx]

**Additional file 8** Positions of the blocks on *B. nigra* LGs in comparison to *B. rapa* genome

|  |  | **LF** | **MF1** | **MF2** |
| --- | --- | --- | --- | --- |
| A | AT1G01560_AT1G02190 | Bra033253_Bra033213_A10 | Bra030497_Bra030497_A08 | Bra032640_Bra032670_A09 |
|  | AT1G02220_AT1G07630 | Bra033301_Bra015596_A10 **B4** | Bra030498_Bra030695_A08 **B7** | Bra032614_Bra031587_A09 **B8** |
|  | AT1G07640_AT1G19330 | Bra018693_Bra025729_A06  **B6** | Bra030696_Bra016509_A08 | Bra031588_Bra031076_A09 |
| B | AT1G19850_AT1G21910 | Bra025775_Bra017957_A06 | Bra016492_Bra016401_A08 | Bra012190_Bra012297_A07 |
|  | AT1G21920_AT1G24210 | Bra031352_Bra024644_A09 | Bra016400_Bra016318_A08 | Bra012298_Bra012416_A07 |
|  | AT1G24220_AT1G27220 | Bra032811_Bra024646_A09 | Bra010955_Bra016317_A08 **B7** | Bra030032_Bra012417_A07 |
|  | AT1G27240_AT1G29020 | Bra032813_Bra032925_A09  **B6** | Bra010954_Bra010851_A08 | Bra030033_Bra030121_A07 |
|  | AT1G29030_AT1G30970 | Bra032290_Bra023153_A09 | Bra010850_Bra010764_A08 | Bra030122_Bra014897_A07 **B7** |
|  | AT1G30972_AT1G31750 | Bra023154_Bra023199_A09 | Bra038403_Bra038434_A08 | Bra014898_Bra014913_A07 |
|  | AT1G31760_AT1G36240 | Bra023200_Bra028085_A09 | Bra038435_Bra036766_A08 **B1** | Bra033887_Bra034400_A05 |
| C | AT1G43600_AT1G47260 | Bra036926_Bra040713_Scaffold000123 | Bra014019_Bra014069_A08 | Bra032192_Bra032220_A05 |
|  | AT1G47270_AT1G47946 | Bra015624_Bra015599_A9 **B6** | Bra014070_Bra014098_A08 **B1** | Bra032233_Bra032228_A05 |
|  | AT1G47960_AT1G56120 | Bra018695_Bra038035_A06 **B6** | Bra014099_Bra030816_A08 | Bra032221_Bra037091_A05 |
| D | AT1G56530_AT1G62045 | Bra017901_Bra017885_A03 | Bra035468_Bra028377_A01 **B1** | Bra027924_Bra027067_A09 |
|  | AT1G62060_AT1G63770 | Bra034461_Bra004106_scaffold | Bra036663_Bra027789_A09 | Bra027066_Bra027663_A09 **B6** |
| E | AT1G65040_AT1G67210 | Bra004109_Bra004209_A07 | Bra022497_Bra034014_A02 | - |
|  | AT1G67220_AT1G79720 | Bra004210_Bra035139_A07 **B4**  **B7** | Bra034013_Bra008428_A02  **B2** | Bra004099_Bra003596_A07  **B7** |
|  | AT1G79760_AT1G80420 | Bra035206_Bra035184_A07 | Bra008503_Bra008474_A02 | Bra003527_Bra003565_A07 **B4** |

| F | AT3G01040_AT3G02070 | Bra039149_Bra039109_A05 | Bra021479_Bra040520_A01 | Bra001025_Bra000980_A03 |
| --- | --- | --- | --- | --- |
|  | AT3G02080_AT3G23150 | Bra039150_Bra033888_A05 **B5** | Bra021478_Bra023756_A01 **B1** | Bra001028_Bra001904_A03 **B1** |
|  | AT3G23160_AT3G25520 | Bra014914_Bra015128_A07 | Bra023755_Bra028373_A01 | Bra001905_Bra013215_A03 |
| G | AT2G05170_AT2G10931 | Bra015164_Bra015187_A07 | - | Bra013178_Bra013160_A03 **B8** |
| H | AT2G10940_AT2G17090 | Bra015198_Bra002020_A07 | Bra037196_Bra037283_A09 | Bra013156_Bra013035_A03 |
|  | AT2G17110_AT2G19470 | Bra002021_Bra039003_A07 | Bra037281_Bra036721_A09 **B6** | Bra009665_Bra024449_A06 **B8** |
|  | AT2G19480_AT2G20900 | Bra039004_Bra036507_A07 **B7** | Bra036720_Bra036670_A09 | Bra031083_Bra031147_A09 |
| I | AT2G20920_AT2G26660 | Bra031151_Bra007748_A09 **B8** | Bra030326_Bra034308_A04 | Bra000588_Bra000539_A03 **B3** |
|  | AT2G26670_AT2G29090 | Bra012054_Bra028390_A07 **B4** | Bra034312_Bra039963_A04 **B5** | Bra000561_Bra000478_A03 |
| J | AT2G31040_AT2G40420 | Bra018282_Bra004963_A05 | Bra021690_Bra017005_A04 | Bra022823_Bra000184_A03 |
|  | AT2G40430_AT2G46130 | Bra004567_Bra004962_A05 **B4** | Bra017004_Bra039289_A04 **B5** | Bra000185_Bra000413_A03 **B3** |
|  | AT2G46140_AT2G47730 | Bra004566_Bra004434_A05 | Bra039288_Bra021440_A04 | Bra000414_Bra000460_A03 |
| K | AT2G01250_AT2G03750 | Bra024895_Bra025130_A06 **B8*** | Bra014334_Bra026541_A02 **B6** | Bra017461_Bra017375_A09  **B6** |
| L | AT3G25855_AT3G29770 | Bra025166_Bra025404_A06 **B8** | Bra036363_Bra033110_A02 **B6** | Bra017345_Bra036205_A09 **B1 +B6** |
| M | AT3G43740_AT3G49970 | Bra019444_Bra017958_A03 **B3** | Bra037606_Bra029985_A01  **B2** | Bra019453_Bra019606_A06 **B3 + B8** |
| N | AT3G50950_AT3G51860 | Bra036844_Bra006881_A09 | Bra038875_Bra038910_A01 | Bra012876_Bra012833_A03 **B2** |
|  | AT3G51870_AT3G52760 | Bra006882_Bra006945_A09 **B3** | Bra033476_Bra019650_A04 | Bra012832_Bra012799_A03 **B8** |
|  | AT3G52770_AT3G62790 | Bra006946_Bra007701_A09 | Bra019649_Bra014392_A04  **B4** | Bra028531_Bra003510_A07 **B4** |
| O | AT4G00030_AT4G00780 | Bra037309_Bra037358_A09 | Bra000979_Bra000955_A03 | Bra008511_Bra008532_A02 |
|  | AT4G00800_AT4G01380 | Bra037410_Bra037362_A09 | Bra000929_Bra000954_A03 **B3** | Bra008544_Bra008533_A02 **B2** |
|  | AT4G01390_AT4G03728 | Bra037411_Bra034225_A09 **B3** | Bra000928_Bra000815_A03 | Bra008545_Bra018514_A02 |
|  | AT4G03740_AT4G04955 | Bra029471_Bra029537_A09 | Bra000780_Bra000810_A03 | Bra018492_Bra018508_A02 |
| P | AT4G08690_AT4G12070 | Bra037874_Bra029437_A09 **B3** | Bra000655_Bra000760_A03 **B3** | Bra033210_Bra033122_A02 **B2** |
| Q | AT5G23030_AT5G23270 | Bra013010_Bra013029_A03 | Bra029357_Bra029365_A02 **B2** | Bra026507_Bra026507_A09 |
|  | AT5G23280_AT5G28470 | Bra009667_Bra009996_A06 **B8** | Bra029366_Bra020609_A02 **B6** | Bra026506_Bra036119_A09 |
|  | AT5G28490_AT5G28885 | Bra009997_Bra010012_A06 | Bra020610_Bra020621_A02 | Bra036134_Bra036126_A09 **B1** |
| R | AT5G01240_AT5G02000 | Bra009636_Bra009646_A10 | Bra005693_Bra005657_A03 | Bra028894_Bra028918_A02 |
|  | AT5G02010_AT5G23010 | Bra009621_Bra002448_A10 **B8** | Bra005701_Bra006645_A03 **B3** | Bra028883_Bra020228_A02  **B2** |
| S | AT5G33210_AT5G37810 | Bra028101_Bra028151_A04 | Bra039484_Bra039503_A05 | Bra038948_Bra035522_A08 **B1** |
|  | AT5G37830_AT5G41900 | Bra028154_Bra025457_A04 **B5** | Bra028392_Bra028514_A07 **B4** | Bra025434_Bra030327_A04 |
| T | AT4G12750_AT4G13985 | Bra032672_Bra033456_A04 | - | Bra034911_Bra040347_A08 **B1** |
|  | AT4G13990_AT4G14140 | Bra032753_Bra032760_A04 **B4** | - | Bra038960_Bra038444_A08 |
|  | AT4G14145_AT4G14490 | Bra032763_Bra033481_A04 | - | Bra010747_Bra010762_A08 **B7** |
|  | AT4G14500_AT4G15160 | Bra036864_Bra039563_A01 | - | Bra039861_Bra038089_A08 |
|  | AT4G15180_AT4G16160 | Bra039562_Bra033542_A01 **B2** | Bra012769_Bra012736_A03 **B8** | Bra038088_Bra038039_A08 |
|  | AT4G16162_AT4G16143 | Bra033544_Bra033534_A01 | Bra012735_Bra012741_A03 | Bra021082_Bra038043_A08 |
| U | AT4G16250_AT4G24180 | Bra033549_Bra013773_A01 | Bra012725_Bra019243_A03 **B8** | Bra021080_Bra010549_A08 **B1** |
|  | AT4G24190_AT4G28405 | Bra013774_Bra026259_A01 | Bra019235_Bra024205_A03 | Bra010500_Bra010369_A08 **B7** |
|  | AT4G28410_AT4G35320 | Bra040157_Bra011606_A01 | Bra024204_Bra017708_A03 | Bra010368_Bra020811_A08 |
|  | AT4G35335_AT4G36120 | Bra011607_Bra011663_A01 **B2** | Bra017709_Bra017748_A03  **B8** | Bra010537_Bra010501_A08 |
|  | AT4G36130_AT4G38170 | Bra011664_Bra011805_A01 | Bra017749_Bra017884_A03 | Bra010550_Bra010636_A08 |
|  | AT4G38190_AT4G38770 | Bra011900_Bra011893_A01 | Bra033554_Bra033575_A06 | Bra010746_Bra010727_A08 |
| V | AT5G42130_AT5G47780 | Bra033636_Bra024917_A06 **B8** | Bra021948_Bra022156_A02 **B6** | Bra027974_Bra017467_A09 **B6** |
| W | AT5G47820_AT5G50750 | Bra037951_Bra037925_A10 | Bra000592_Bra000630_A03 **B3** | Bra020729_Bra022524_A09 **B1** |
|  | AT5G50760_AT5G60800 | Bra035710_Bra002450_A10 **B8** | Bra022763_Bra006647_A03 **B3** | Bra022525_Bra020229_A02 **B2** |
| X | AT5G60810_AT5G61760 | Bra013006_Bra012923_A03 **B8** | Bra029354_Bra029318_A02 | Bra035957_Bra035915_A09 |
|  | AT5G61770_AT5G65925 | Bra019611_Bra024431_A06 **B4** | Bra029309_Bra031843_A02 | Bra035914_Bra037833_A09 **B1** |
|  | AT5G65930_AT5G67385 | Bra012055_Bra012158_A07 | Bra031800_Bra031830_A02 | Bra037195_Bra037116_A09 |
