# Supplementary material for: RNA-seq based SNPs for mapping in Brassica juncea (AABB): synteny analysis between the two constituent genomes A (from B. rapa) and B (from B. nigra) shows highly divergent gene block arrangement and unique block fragmentation patterns
Source: BMC Genomics. 2014 May 23;15(1):396. doi: 10.1186/1471-2164-15-396 (PMC4045973; doi:10.1186/1471-2164-15-396)
Supplement: Supplementary file 9 — Additional file 9: Comparison of the number of SNPs identified in different lines of B. rapa and in the A and B genomes of B. juncea. The number of SNPs identified between various lines of B. rapa are taken from our earlier study [22]. B. juncea data were generated in this study. (DOCX 14 KB) [file 12864_2013_6090_MOESM9_ESM.docx]

**Additional file 9** Comparison of the number of SNPs identified in different lines of *B. rapa* and in the A and B genomes of *B. juncea*

| Brassica species | Comparison between lines | Total number of genes expressed | Gene models with SNP differences | Total Number of SNPs |
| --- | --- | --- | --- | --- |
| *B. rapa* | Chiffu vs Tetra | 41,173/28,715 | 15,400 | 249,671 |
| *B. rapa* | Chiffu vs Candle | 41,173/23639 | 16,179 | 266,349 |
| *B. rapa* | Chiffu vs YSPB-24 | 41,173/22996 | 14,505 | 231,259 |
| *B. rapa* | YSPB-24 vs Tetralocular | 22,996/28,715 | 5,117 | 28,768 |
| *B. juncea* | Heera A genome vs Varuna A genome | 21,212/21,046 | 9,035 | 85,437 |
| *B. juncea* | Heera B genome vs Varuna B genome | 19,195/19,329 | 5,921 | 50,437 |

* Chiffu gene model number is taken from the BRAD database. Except Chiffu all the other *B. rapa* data is taken from the transcriptome sequencing of different *B. rapa* lines [22]. *B. juncea* data is generated from this study
